# An endometrial gene expression signature accurately predicts recurrent implantation failure after IVF

**DOI:** 10.1038/srep19411

**Published:** 2016-01-22

**Authors:** Yvonne E. M. Koot, Sander R. van Hooff, Carolien M. Boomsma, Dik van Leenen, Marian J. A. Groot Koerkamp, Mariëtte Goddijn, Marinus J. C. Eijkemans, Bart C. J. M. Fauser, Frank C. P. Holstege, Nick S. Macklon

**Affiliations:** 1 Department of Reproductive Medicine and Gynaecology, University Medical Center Utrecht, Utrecht, The Netherlands; 2 Molecular Cancer Research, University Medical Center Utrecht, Utrecht, The Netherlands; 3 Center for Reproductive Medicine, Academic Medical Center, University of Amsterdam, Amsterdam, The Netherlands; 4 Julius Center for Health Sciences and Primary Care, University Medical Center Utrecht, Utrecht, The Netherlands; 5 Human Development and Health, Faculty of Medicine, University of Southampton, Southampton, United Kingdom

## Abstract

The primary limiting factor for effective IVF treatment is successful embryo implantation. Recurrent implantation failure (RIF) is a condition whereby couples fail to achieve pregnancy despite consecutive embryo transfers. Here we describe the collection of gene expression profiles from mid-luteal phase endometrial biopsies (n = 115) from women experiencing RIF and healthy controls. Using a signature discovery set (n = 81) we identify a signature containing 303 genes predictive of RIF. Independent validation in 34 samples shows that the gene signature predicts RIF with 100% positive predictive value (PPV). The strength of the RIF associated expression signature also stratifies RIF patients into distinct groups with different subsequent implantation success rates. Exploration of the expression changes suggests that RIF is primarily associated with reduced cellular proliferation. The gene signature will be of value in counselling and guiding further treatment of women who fail to conceive upon IVF and suggests new avenues for developing intervention.

Despite advances in assisted reproductive techniques (ART), the majority of IVF attempts still do not result in a successful pregnancy. Failure of implantation of apparently morphologically sound embryos now represents the major limiting step in IVF success. A significant proportion of couples undergoing IVF experience recurrent implantation failure (RIF), a devastating occurrence for patients in which serial transfers of high quality embryos fail to result in a pregnancy^1^. The perceived need among clinicians and patients to intervene to address RIF successfully has led to the introduction of numerous empirical and thus far ineffective adjuvant interventions. Successful management of this frustrating and costly complication of IVF requires a greater understanding both of the underlying mechanisms, and of the individual prognosis for ultimate success when RIF occurs.

RIF has been defined as the absence of implantation after three or more transfers of high quality embryos or after placement of 10 or more embryos in multiple transfers^2^,^3^. Studies of the probability of a systemic underlying cause for implantation failure in these patients, rather than simply a chance effect, indicate that an underlying aetiology is likely to exist in most RIF patients^3^,^4^,^5^,^6^,^7^,^8^. Multiple aetiologies for implantation failure have been proposed but until recently the primary focus has been on the embryo, and in particular the impact of aneuploidy^5^,^9^,^10^. Maternal factors may also contribute, and the clinical approach to investigating RIF now involves the exclusion of thrombophilic gene mutations, autoimmune conditions and uterine anomalies. However, in the majority of cases no clear cause can be identified^3^,^7^,^11^,^12^. In recent years it has become apparent that constitutive endometrial dysfunction could represent an important contributor to this condition^11^,^13^,^14^.

Recent studies have advanced our understanding of the mechanisms which renders the human endometrium receptive for a limited period in the mid-luteal phase of the menstrual cycle, and of the biological significance of this putative ‘window of receptivity’^11^,^15^,^16^,^17^. Although no single, clinically relevant morphological, molecular or histological marker capable of indicating endometrial receptivity has been identified, global transcriptomic and secretomic analyses of human endometria are now providing us with novel insights into patterns of gene and protein expression which characterise the receptive endometrium.

Endometrial gene expression has been shown to be sensitive to cyclical hormonal regulation^16^,^18^,^19^,^20^,^21^,^22^, ovarian stimulation for IVF^23^,^24^,^25^ and to be disrupted in the presence of gynaecological pathologies such as endometriosis^26^,^27^ or during the use of an intrauterine device^28^. Genome-wide analyses have yielded insight into mRNA expression changes during the natural endometrium cycle^29^ and growing evidence supports the concept of a receptive gene expression profile^22^,^30^,^31^, which may be disrupted in patients experiencing RIF^13^,^14^,^22^. The potential value of identifying a gene expression profile predictive of RIF is considerable as this would not only guide prognosis, but inform appropriate and effective therapeutic intervention^14^,^32^.

Thus far studies that directly compare endometrial mRNA expression in RIF patients with controls, have been limited^13^ and not subject to validation on an independent cohort, a requirement to draw any firm conclusions. This is in part due to the challenge of recruiting sufficient participants willing to undergo an endometrium biopsy. In a different approach, a selected subset of genes with altered expression in the endometrium during the natural cycle of healthy women^33^ has been studied in RIF patients, but a significant association with RIF was not found for this particular subset of genes^32^. In the study presented here, we sought to rigorously determine whether endometrial gene expression differs between women with RIF and controls, with the goal of identifying and validating the endometrial gene expression signature associated with RIF.

## Results

### Patients and samples

Mid-luteal phase endometrial biopsies were obtained from 43 women with RIF, and 72 controls, i.e. women who gave live birth after IVF/ICSI. Clinical characteristics of the RIF patients and controls are described in Table 1. No significant differences in age, BMI, smoking, number of women with primary infertility and cause of infertility were identified between controls and RIF patients. As a corollary of selection criteria, the mean number of embryo transfers carried out before endometrial sampling was greater in RIF patients than controls (5.3 vs. 2.4), together with the mean number of transferred embryos (7.8 vs. 3.1). No more than two embryos were inserted in the uterine cavity per transfer and for patients under the age of 36, only single embryo transfers were performed during the first two IVF or ICSI attempts. The mean implantation rate per transferred embryo for RIF patients was 3.2%, compared with 62.7% in the control group. Six patients in the RIF group reported having a live birth before embarking on further unsuccessful IVF/ICSI treatment. Two derived from spontaneous conception and four from multiple ART treatments. All six subjects met the criteria for inclusion in the RIF group after failing multiple IVF/ICSI treatments trying to conceive a second child. Nineteen patients (26%) from the control population had delivered two live births and four patients (6%) delivered three live births after ART treatment prior to inclusion in the study.

### Expression profiles

mRNA expression profiles were successfully obtained from all samples. Principle component analysis (PCA) indicated batch effects and an effect related to slight variations in the biopsy timing (Methods, Supplementary Fig. 1 and Supplementary Table 1). The batch effects were consistent with the batch-wise processing of samples and were successfully removed by statistical modeling (Methods, Supplementary Fig. 1 and Supplementary Table 1). A minor batch effect related to the medical center where the biopsy was performed was not removed because the number of samples was too small to accurately model the effect (n = 9 for the Academic Medical Center (AMC) versus n = 106 for the University Medical Center Utrecht (UMCU)). None of the other variables (age, BMI, smoking, nature of infertility and nature of treatment) showed a significant effect on the gene expression profiles (Supplementary Fig. 2 & Supplementary Table 2).

### Gene signature based prediction

As described in detail in the Methods section, samples were randomly assigned into a signature discovery set (n = 81) and an independent validation set (n = 34), keeping the ratio of RIF patients to controls similar. Iterative rounds of cross-validation (Supplementary Fig. 3) were applied within the signature discovery set to find genes capable of distinguishing RIF patients from controls. The use of cross-validation reduces the risk of over-fitting on the signature discovery set. Each iteration results in a separate gene set. All genes were then ranked according to how frequently they were present in the separate gene sets. Selecting all genes with a frequency of 5% or higher, results in a 303 gene signature (Supplementary Table 3). The 303 genes selected by the cross-validation approach are those most suitable for distinguishing between the two groups and were therefore subsequently employed in a support vector machine (SVM) classifier built using the entire discovery set (Supplementary Fig. 3 and Methods).

**Figure 1:**
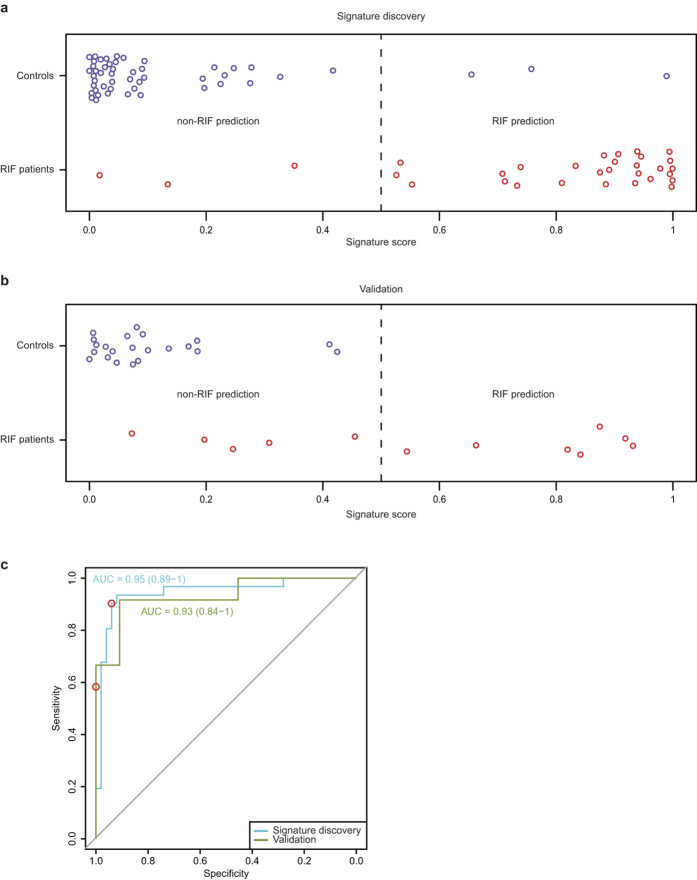
Signature discovery and validation. This figure shows SVM classifier results on the signature discovery (**a**) and validation (**b**) sets. RIF signature genes were first determined on the signature discovery sample set by 100 rounds of cross-validation (Supplementary Fig. 3, Methods). Using these 303 genes, (panel **a**) shows SVM classifier scores for the signature discovery set (patients: red, controls: blue) using leave one out cross validation. Samples with a score below 0.5 are predicted to be controls, those with a score of 0.5 or higher are predicted to be RIF patients (the threshold is shown as a dotted line). (Panel **b**) shows all samples from the validation set scored based on the SVM classifier trained on all samples in the signature discovery set. (Panel **c**) shows the ROC curves for the results shown in A (blue line) and B (green line). The Area Under the Curve (AUC) with the 95% CI is shown next to the curves. The dots indicate the point of the ROC curve that corresponds with the threshold used for classification (0.5)

Figure 1a shows the classification of samples in the discovery set using the 303-gene classifier. The accuracy of the RIF prediction (PPV) was 90% with a sensitivity of 90% (Table 2). Most importantly, application to the independent validation set confirms the signature’s ability to distinguish RIF patients from controls (Fig. 1b). All samples classified as RIF were indeed RIF patients (PPV = 100%) with a sensitivity of 58%. The non-RIF classification was accurate in 81% of cases (Table 2). The areas under the ROC curves (Fig. 1c and Supplementary Fig. 4) confirm that gene expression based classification in general (Supplementary Fig. 4), and the 303-gene signature in particular (Fig. 1c), can robustly classify RIF patients.

An obvious difference between RIF patients and controls is the frequency of successful implantations. Differences in gene expression between RIF patients and controls could therefore be due to the effects of previous pregnancy rather than reflecting a more direct link to implantation failure. To investigate this, the 303-gene expression signatures of patients with and without a previous pregnancy were compared. No difference was found (Supplementary Fig. 5), ruling out that the RIF status prediction reported here is confounded by (the absence of) previous pregnancies.

### Functional analysis of RIF endometrial gene expression

**Figure 2:**
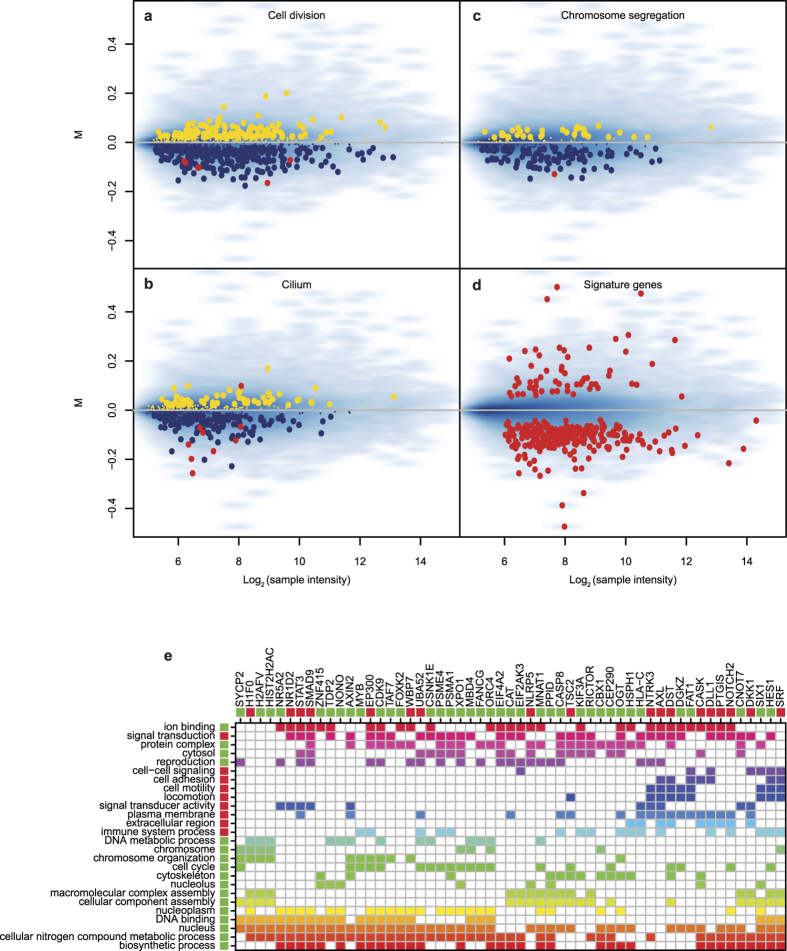
Gene set enrichment analysis. (Panels **a** to **d**) show the gene expression of RIF patients compared against controls (log2 fold change or M) and the average expression across all samples (log2 sample intensity). (Panels **a** to **c**) each focus on an example of a Gene Ontology term which was found to be significant in GSEA. Genes in the GO term up-regulated in RIF patients are shown in yellow, genes down-regulated in blue. Shown in red are the genes which are also part of the 303-gene signature. Genes that are not part of the GO term are shown as a blue density map where darker blue indicates higher gene density. (Panel **d**) shows all the genes of the gene signature. (Panel **e**) shows a selection of 55 genes from the 303-gene signature and 26 GO terms in which they are involved. The genes selection was based on the number of GO terms associated to the gene (>6), the GO terms were selected based on their statistical significance in the GSEA and the number of genes associated (>6). The selection was performed for visual clarity. The bars below the gene names and to the right of the GO terms indicates whether a gene/GO term is up-regulated in RIF patients (red) or down-regulated (green). All other colours are solely for visualization purposes and do not indicate strength of association. The rows and columns are clustered based on Euclidean distance. For an unfiltered version see Supplementary Fig. 7.

Besides classification of patients, an additional benefit of gene expression analyses is the potential to shed light on factors underlying a particular condition. A striking characteristic of the RIF signature genes is that there are many more genes with decreased expression (81%, Fig. 2d). Gene set enrichment analysis (GSEA) of the entire expression-profiles using Gene Ontology (GO) slim categories indicates more specifically the various cellular processes and structures differentially affected in RIF patients versus controls (Table 3). Most striking is the down-regulation in RIF patients of genes involved in cell cycle regulation and cell division (Table 3, Fig. 2a), indicative of a reduced rate of cellular proliferation. Besides reduced expression of many genes involved in general proliferative processes (Table 3), the RIF endometrium transcriptome also shows reduced expression of genes involved in cytoskeleton and cilia formation (Table 3, Fig. 2b). The latter is of interest given the presence of ciliated cells during the implantation window of healthy women^34^. A previous study indicated that a considerable fraction of genes down-regulated in RIF patients are estrogen dependent^13^. Here, no strong indication of estrogen dependent down-regulation of gene expression is found in RIF patients (Supplementary Fig. 6). A possible explanation lies in the definition of ‘estrogen dependent’. The previous study^13^ referred to the effect of estrogen depletion on gene expression in general^35^, whereas our analysis (Supplementary Fig. 6) applies the specific Gene Ontology term ‘response to estrogen’. Notably, Bourdeau *et al.* demonstrated the association between estrogen depletion and cell cycle progression^35^. The differentially regulated genes are therefore likely to be cell cycle related and not necessarily directly regulated by estrogen. This is consistent with our observation of a down-regulation of cell proliferation in RIF patients. Similar to the number of up-regulated genes in the RIF signature, the number of enriched GO categories with increased expression is more limited, but do exhibit common features: processes involved in extracellular organization and cell motility (Table 3).

The gene set enrichment analysis was performed on the entire transcriptomes of RIF patients and controls. For individual genes from the RIF signature, Fig. 2e shows their representation within the various functional classes (see Supplementary Fig. 7 for the complete overview of all signature genes). The individual signature genes are also listed in Supplementary Table 3. Another feature of the RIF signature is the high proportion of transcription factors, indicated by enrichment of the GO term DNA binding (Table 3). Besides many uncharacterized DNA binding proteins, the signature gene set also contains established transcription factors such as the forkhead transcription factor FOXK2, family members of which have previously been implicated in ovary development and function^36^,^37^.

### Further expression-based patient stratification

**Figure 3:**
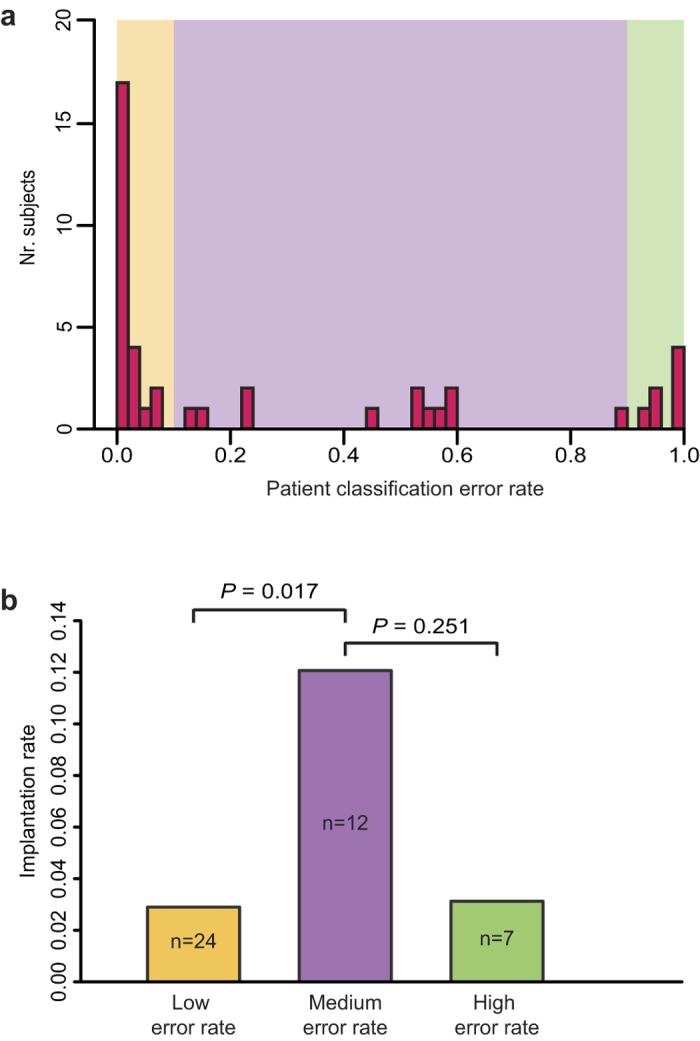
Patient stratification. (Panel **a**) shows the distribution of the RIF patient classification error rates (n = 43). The error rate is the ratio of misclassifications to number of classification attempts (see Methods for details). The coloured rectangles denote patient groups with similar error rates (low < 0.1, medium ≥ 0.1 & ≤ 0.9, high > 0.9). (Panel **b**) shows the IVF implantation rate for the three aforementioned patient groups. Implantation rate is defined as implantations per embryo transfer (prior to the biopsy) and includes all outcomes: i.e. biochemical pregnancy, miscarriage, live birth. *P* was calculated using a two-sided Fisher’s exact test comparing combined outcomes of all IVF cycles.

The gene expression classifier yields a binary result, classifying subjects as either RIF or control. Some RIF patients are incorrectly classified (Fig. 1a,b). To investigate whether this was a random occurrence influenced by the composition of the final gene set, the iterative signature discovery procedure (Supplementary Fig. 3) was repeated using all patients and controls. Each round of the signature discovery procedure consists of predicting patients and controls using a different gene set (Methods). By assessing the predictions for each round, a robust classification error rate was determined for each patient (Fig. 3a). Strikingly, one group of RIF patients is almost always misclassified (Fig. 3a, right, > 90% error rate). This indicates that misclassification is not a random event and suggests that this subpopulation actually represents a distinct class of RIF patients for which the underlying cause of RIF is different and not represented in the endometrial gene expression pattern.

Analysis of the classification error rate (Fig. 3a) also indicates two other groups: RIF patients with a low classification error rate (Fig. 3a, left, < 10% error rate) and an intermediate group, more difficult to classify by gene expression (error rate between 10% and 90%). To determine whether this stratification has any clinical relevance, the implantation success rate per IVF cycle was determined for the three groups (Fig. 3b). By definition all three RIF groups have a low implantation success rate. Interestingly, RIF patients with an intermediate classification error rate have a significantly higher implantation success rate compared to the RIF patients with a low classification error rate (Fig. 3b). The higher implantation success rate for the intermediate group fits with the idea that this group presents an intermediate expression-based RIF phenotype as judged by the classification error rate. This further stratification strengthens the conclusion that expression-based classification is clinically relevant. The RIF patients with the highest classification error rate (>90%) seem to have a low implantation success rate (Fig. 3b). Although this would agree with their status as a distinct group of patients, suffering from RIF but without presenting an aberrant expression signature, the difference in implantation success is not statistically significant compared to either of the two other groups.

## Discussion

Studies of the impact of the endogenous and exogenously manipulated hormonal milieu on endometrial gene expression^25^,^29^ have indicated the susceptibility of the endometrium to disruption by such factors. An early genome-wide study, directly comparing RIF patients with controls has strengthened this^13^. However, there has been a lack of large human studies allowing for the robust determination of a RIF-associated gene expression signature while also incorporating an independent validation. In this study, the number of samples has been sufficiently high to determine a gene signature and show on an independent set that the signature is robustly capable of distinguishing between women experiencing RIF and IVF controls.

A gene signature that predicts RIF with a high PPV offers the ability to identify patients whose chances of a successful pregnancy are very small, which is of clear value in counselling patients as to the wisdom of investing further time and effort and money in further treatment. Moreover, the strong negative predictive value (NPV) of 81% indicates that, given a non-RIF expression profile, the chance of an endometrial factor impeding treatment success is small. This would suggest a positive outlook for continued treatment, especially if embryo quality can be improved, by using donated oocytes for example. Discerning whether RIF in a particular patient reflects impaired endometrial or embryo quality, a perturbed dialog between the embryo and the endometrium, or simply a chance event, remains challenging. However, the different predictive accuracies observed for RIF patients (Fig. 3a), whereby some are almost always correctly classified while others are not, enables those with a significant endometrial factor to be identified. Those who are ‘misclassified’ and therefore more closely resemble an expression profile of a healthy, receptive endometrium, are likely to have a significant embryo quality issue. However, a sizeable number of patients fall in-between the two groups, implying a non-binary distinction between refractory and receptive endometrium. It is interesting to observe that this group of patients had a better IVF implantation rate compared to the patients with a clear RIF expression signature. This suggests that the strength of the RIF-associated expression profile is correlated with the severity of the RIF phenotype.

Gene function analysis shows a strong skew towards down-regulation of processes in RIF patients. This is consistent with a previous study of RIF^13^ that also reported enrichment for cell cycle associated gene function within the set of down-regulated genes. Interestingly, an endometrium lagging behind the normal phase of proliferative and secretory events has recently been put forward as a cause of implantation failure^32^, and a gene expression test designed to identify this has recently been commercialized (Endometrial Receptivity Array (ERA), iGenomix, Valencia, Spain)^32^. The genes present on the ERA were determined from studies of the endometrial cycle in healthy individuals^25^,^22^ and the test does not distinguish between patients with RIF and controls^32^. In contrast, the classifier presented here was determined directly from differential expression between RIF patients and controls, and thus performs well in distinguishing between these two groups (Fig. 1).

The invasive nature of endometrial biopsy required to obtain tissue for testing precludes its use in a treatment cycle. However, clinical application of the 303-gene signature could be practical in a non-treatment cycle after multiple failed IVF treatments, when a RIF diagnosis has already been established. A signature-based diagnostic test to investigate the contribution of an endometrial factor in implantation failure would be a meaningful enhancement to the current limited clinical management strategies. However, perhaps the greatest clinical benefit is to be found in diagnosing receptive or refractory endometrium prior to the start of IVF/ICSI treatment. Up to one third of couples presenting with infertility have no cause identified by routine investigations^9^. However, at present there is no validated test of endometrial receptivity. It can be proposed that couples presenting with infertility would benefit from a test of endometrial receptivity as part of their initial investigations, in order to identify whether there is a significant endometrial factor which may affect their chances of conceiving either spontaneously or by IVF treatment. Clearly, validation of the 303-gene predictor in prospective cohorts of IVF/ICSI patients would be required.

## Methods

### Study design and tissue collection

The study consists of two serial cohorts. In each cohort women experiencing RIF and fertile IVF/ICSI controls participated. The study was approved by the Medical Review Ethics Committee of the University Medical Center Utrecht and the Medical Review Ethics Committee of the Academic Medical Center and performed in accordance with the approved guidelines. Written informed consent was obtained from all participating subjects.

All participants had undergone IVF/ICSI treatment in two tertiary referral academic hospitals. They were aged ≤38 years at time of biopsy (one patients was 39 and 5 days at biopsy, 38 at diagnosis RIF), had regular menstrual cycles of 25–35 days, and did not use oral contraceptives or an intra-uterine device.

IVF and ICSI treatments were performed according to local protocols. Treatments were started after at least one year of trying to conceive spontaneously. All included women had undergone routine fertility investigations, and all had an indication for IVF/ ICSI treatments according to the Dutch Society of Obstetrics and Gynaecology guidelines^38^. Women underwent controlled ovarian hyperstimulation with recombinant FSH (Puregon, MSD, The Netherlands/ Gonal-F, Merck Serono, Germany) or HMG (Menopur, Ferring, The Netherlands). To prevent a premature LH surge, pituitary suppression was achieved by co-treatment with the GnRH agonist triptorelin (Decapeptyl, Ferring, The Netherlands) or a GnRH antagonist (Orgalutran, MSD, The Netherlands/ Cetrotide, Merck Serono, Germany). Follicular maturation was induced by 10,000 IU urinary hCG (Pregnyl, MSD, The Netherlands). Cumulus oocyte complexes were retrieved by transvaginal ultrasound-guided follicle aspiration 36 hours after hCG injection. Oocytes were inseminated with 10,000–15,000 progressively motile spermatozoa (IVF) or injected with a single spermatozoon 2–4 hours after follicle aspiration (ICSI). Embryo transfer was performed 3–4 days after oocyte retrieval using a Wallace catheter (Smiths Medical, USA). Luteal phase support was performed by intravaginal progesterone 400–600 mg/day (Utrogestan, Besins, Belgium).

Supernumerary good quality embryos were frozen on day of the embryo transfer using a slow freeze protocol. Frozen-thawed embryos were transferred in ultrasound monitored natural cycles after ovulation induction with 5,000 IU hCG (Pregnyl), or in artificial cycles using estradiol (Progynova, Bayer Health Care Schering, Germany) and intravaginal progesterone (Utrogestan).

RIF was defined as ≥3 failed IVF or ICSI treatments or transfer of ≥10 embryos without the occurrence of a pregnancy (including frozen-thawed cycles). A pregnancy was defined as a positive HCG serum test or a positive at home urine test 14 days after embryo transfer.

Women meeting the definition for RIF were excluded if implantation failure could be explained by other factors; i.e. poor embryo quality (defined as less than 8 cell embryonal stage at day 3 after oocyte retrieval, or less than 12 cell stage at day 4), poor ovarian response (defined as <4 oocytes retrieved on adequate ovarian stimulation), or known disturbances in the uterine cavity or endometrial pathology, such as uterine anomalies, hydrosalpinx, or evidence of endometriosis (including endometriomas detected by ultrasound or disease diagnosed by laparoscopy). All RIF patients were screened for relevant inherited and acquired thrombophilias and abnormalities in glycosylated haemoglobin (Hb_A1c_) and thyroid-stimulating hormone levels, and excluded when results were aberrant.

The control group consisted of healthy women who had conceived within the first three cycles of IVF or ICSI treatment.

Between June 2006 and November 2007, the first cohort of 22 RIF patients and 23 controls (all ICSI treatment) participated in the University Medical Center Utrecht (UMCU) in Utrecht. Between October 2011 and May 2013, a second cohort was recruited to obtain a sufficient sample size for of signature discovery and validation. This cohort was recruited in the UMCU and the Academic Medical Center (AMC), in Amsterdam, and consisted of 21 RIF patients and 49 controls (26 after ICSI and 23 after IVF treatment).

An endometrial biopsy was scheduled 6 (n = 27) or 7 (n = 71) days after the putative luteinizing hormone (LH) surge in a natural (non-treatment) cycle in patients and controls, which is considered to be a representative day of the window of implantation. The biopsy was never performed in the first natural cycle after an unsuccessful IVF or ICSI cycle, to eliminate the effect of hormonal influence from the ovarian stimulation treatment. Due to scheduling wishes of the participants, several biopsies were performed late on day 5 (n = 8) or early on day 8 (n = 9) after the LH surge. Urinary LH was monitored by a home LH ovulation predictor kit (Ovulady, Clindia Benelux, The Netherlands). Biopsies were collected using an endometrium sampling device (Endobiops Standard CH9, Gynotec, The Netherlands) under sterile conditions. The sample was snap frozen in liquid nitrogen and stored at −80 °C until use.

### Gene expression profiling

#### RNA isolation

Total RNA was isolated from individual tissue samples using Trizol reagent (Invitrogen) following the manufacturer’s protocol (including optional centrifugation), followed by a purification using the RNeasy Mini Kit (Qiagen) and a DNAse treatment using the Qiagen DNA-free kit. The yield and quality of total RNA was checked by spectrophotometry and by the Agilent Bioanalyser (Agilent, Belgium). All 115 samples showed good RNA quality, with clear 18 S and 28S ribosomal bands and RNA integrity numbers (RIN) between 5 and 10.

#### Microarray hybridization

For each total RNA sample, two expression profiles were generated in dye-swap. The samples of both patients and controls were compared against a commercial reference (Universal Human Reference RNA catalog #740000, Stratagene). The microarrays were human whole genome gene expression microarrays V2 (Agilent, Belgium) representing 34,127 *H.sapiens* 60-mer probes in a 4 × 44K layout. Probe sequences from this array were re-annotated by BLAST-searching against database version 71.37 at ENSEMBL. cDNA synthesis, cRNA amplification, labeling, quantification, quality control and fragmentation were performed with an automated system (Caliper Life Sciences NV/SA, Belgium), starting with 3 μg total RNA from each sample, all as previously described in detail^39^. Microarray hybridization and washing was with a HS4800PRO system with QuadChambers (Tecan, Benelux) using 1000 ng, 1–2% Cy5 or Cy3 labeled cRNA per channel as described^39^. Slides were scanned on an Agilent G2565BA scanner at 100% laser power, 30% PMT.

#### Data normalization

After automatic data extraction using Imagene 8.0 (BioDiscovery), the mean spot intensities were normalized using quantile normalization^40^ and the two dye-swapped profiles per sample were merged by averaging the fold changes. Quality control checks using principal component analysis (PCA, sva R package^41^) indicated batch effects as well as a correlation between gene expression variation and the time of biopsy relative to the LH surge (Supplementary Fig. 1A & Supplementary Table 1). Both effects were modelled for each gene individually using linear models (limma R package^42^) and the effect estimates were used to transform the data. Because one of the groups consisted of only six samples (“cohort 2, batch 2”), rather than fitting a single model including biopsy timing, batch and sample class, we opted to estimate the biopsy timing effect using all control samples from the first batch of the second cohort (n = 45). This avoided confounding the biopsy timing effect with the batch effect (by only including a single batch) or with the sample effect (by including only control samples). The effects estimates were used to transform the data using LH + 7 as a reference thereby removing the biopsy timing effect (Supplementary Fig. 1B, right panel and Supplementary Table 1). Batch effects were similarly removed. One batch effect was between the two cohorts. The second cohort also separated into two batches which had been processed for expression profiling in separate runs (Supplementary Fig. 1A, B left panels, Supplementary Table 1). The batch effects were estimated using only control samples (n = 72). This avoids confounding the batch effect with the sample effect (by including only control samples). After estimating the batch effect the data was transformed using the first batch of the second cohort as the reference. Supplementary Fig. 1C shows the transformed data used for all subsequent analyses, devoid of major biopsy timing or batch effects (Supplementary Table 1). All microarray gene expression data have been deposited in the public data repositories ArrayExpress (E-MTAB-2591) and GEO (GSE58144).

### Data analysis

#### Signature discovery

For RIF signature discovery, a randomly selected subset of samples (signature discovery set, n = 81, 38% RIF) was created whereby the ratio of RIF patients to controls was kept similar to the full complement of samples. The remaining samples were assigned to the validation set (n = 34, 35% RIF).

For signature discovery, array probes with a low median intensity in the samples (log2 ≤ 6) were filtered out, as were probes with a standard deviation of expression fold-change versus the reference material that was below the median standard deviation of all probes. Both median intensity and standard deviation were based on the signature discovery set measurements. The intensity filtering was performed to eliminate lowly expressed genes, which are inherently more prone to measurement noise. Filtering against low standard deviation eliminates stably expressed genes with little variation among samples. After filtering expression measurements for intensity and standard deviation, 15,502 of the 34,217 initial array probes remained (12,198 of 22,395 unique genes).

Signature discovery consisted of 100 rounds of randomly selecting a training subset (4/5 of all samples) from the signature discovery set (resampling without replacement) which was subsequently used to rank genes based on their potential to differentiate RIF patients from controls (Supplementary Fig. 3). The signal to noise ratio was used as the ranking metric^43^. The 100 top ranked genes were used to build a linear support vector machine (SVM) classifier (e1071 R package^44^) using the training subset as input. The SVM classifier was built with the option to compute class probabilities. We termed the resultant probability estimate the signature score and used a cutoff of 0.5 (equal probabilities for either class) for classification ( < 0.5 control classification, ≥ 0.5 RIF patient classification). The trained SVM classifier was employed to predict the class of the samples not part of the training subset (test subset). This procedure was repeated 100 times recording the genes selected as well as the prediction results used for generating the ROC curve. After 100 rounds, genes were ranked based on the number of times they appeared in the list of 100 top genes and all genes that appeared > 5 times or more were selected into the final gene signature (Supplementary Fig. 3). This final gene signature contained 320 array probes which represented 303 unique genes. For the sake of consistency, this is referred to as the 303-gene signature. Leave-one-out cross-validation was used to test the effectiveness of this gene signature on the signature discovery set (using an SVM classifier).

Finally, as an independent validation of the gene signature, an SVM classifier was built using the full signature discovery set as input and this classifier was employed to predict the class of the samples in the validation set, which had not been used in any of the previous steps to ensure an independent validation. See Supplementary Fig. 3 for a graphical representation of the signature discovery and validation procedures.

ROC curves for classifier performance were calculated and plotted using the pROC R package^45^.

#### Gene function analysis

To explore the processes differentially regulated between RIF patient and controls gene set enrichment analysis (GSEA)^46^ was performed using the generic GO slim subset of Gene Ontology (GO) terms^47^ as gene sets (database version 2013-03-02). First we calculated the t-statistic for every gene by contrasting log2 fold changes of RIF patients against controls. Secondly, for every gene set/GO term, we calculated a gene set *Z*-score, based on the gene specific t-statistics, which enabled calculation of a two-sided *P* for every GO term from the standard normal distribution^48^. Reported *P* values were corrected for multiple testing using Bonferroni correction.

#### Patient Stratification

During the iterative rounds of signature discovery we noticed that RIF patients in the signature discovery set were not all predicted equally well, with some consistently misclassified as controls, while others were correctly classified in most cases. To investigate this, the signature discovery procedure was repeated, only now including all patients and controls (n = 115) and using 1,000 resamplings instead of 100. In each iteration the outcomes of the predictions were recorded and an overall error rate was calculated (false predictions/number of predictions) for every patient. The patients were divided into three groups based on two, arbitrarily chosen, error rate thresholds (0.1 and 0.9).

## Additional Information

**Accession codes:** All microarray gene expression data have been deposited in the public data repositories ArrayExpress (http://www.ebi.ac.uk/arrayexpress/, accession number E-MTAB-2591) and GEO (http://www.ncbi.nlm.nih.gov/geo, accession number GSE58144).

**How to cite this article**: Koot, Y. E. M. *et al.* An endometrial gene expression signature accurately predicts recurrent implantation failure after IVF. *Sci. Rep.* **6**, 19411; doi: 10.1038/srep19411 (2016).

## Supplementary Material

Supplementary Information

## Figures and Tables

**Table 1:** Patient characteristics at time of biopsy.

Characteristic	Value	RIF patients	Controls	*P*
Subjects, n		43	72	
Female age, mean (range)		34.0 (27–38)	34.6 (26–39)	0.204
BMI, mean (range)		23.7 (19–37)	25.0 (19–53)	0.288
Smoking, n (%)		1 (2)	8 (11)	0.087
Primary infertility, %		79	90	0.162
Cause of infertility, n (%)	Andrological	28 (65)	52 (72)	0.110
	Unexplained subfertility	6 (14)	15 (21)	
	Tubal pathology	6 (14)	2 (3)	
	Unknown	3 (7)	3 (4)	
Treatment history before biopsy	Embryo transfers, mean (range)	5.3 (3–12)	2.4 (1–18)	2.9 × 10^−12^
	Transferred embryos, mean (range)	7.8 (3–18)	3.1 (1–30)	2.8 × 10^−13^
	Implantations, mean (range)	0.3 (0–2)	1.4 (1–3)	4.0 × 10^−15^
	Implantation rate, mean %	3	63	< 2.2 × 10^−16^

Age, age at biopsy; BMI, body mass index; Smoking, number of patients who smoked (range: 1–10 cigarettes); Primary infertility, percentage of patients who were nulliparous at entrance of IVF/ICSI treatment (unknown for 1 patient & 3 controls); Implantation rate, implantations/transferred embryos; Day of biopsy, day of endometrial biopsy after positive LH surge; *P* was calculated using the Mann-Whitney test for continuous data and Fisher’s exact test for count data (both two-sided).

**Table 2:** Classification metrics.

Metric	Signature Discovery	Validation
NPV, % (95% CI)	94.0 (83.8–97.9)	81.5 (63.3–91.8)
PPV, % (95% CI)	90.3 (75.1–96.7)	100 (64.6–100)
Sensitivity, % (95% CI)	90.3 (75.1–96.7)	58.3 (32.0–80.7)
Specificity, % (95% CI)	94.0 (83.8–97.9)	100 (85.1–100)
Overall accuracy, % (95% CI)	92.6 (84.8–96.6)	85.3 (69.9–93.6)
*P*	3.83 × 10^−13^	0.0147

NPV, Negative predictive value; PPV, positive predictive value; *P* was calculated using Fisher’s exact test (two-sided); (95% CI) 95 percent confidence interval, calculated using the Wilson method.

**Table 3:** GSEA results.

GO Identifier	Description	*Z*-score	*P*
GO:0005634	nucleus	−14.82	1.36 × 10^−47^
GO:0005886	plasma membrane	12.90	5.73 × 10^−36^
GO:0005694	chromosome	−12.64	1.67 × 10^−34^
GO:0007049	cell cycle	−12.16	6.99 × 10^−32^
GO:0006259	DNA metabolic process	−11.62	4.35 × 10^−29^
GO:0004871	signal transducer activity	11.46	2.79 × 10^−28^
GO:0034641	cellular nitrogen compound metabolic process	−11.06	2.61 × 10^−26^
GO:0005576	extracellular region	10.61	3.62 × 10^−24^
GO:0005815	microtubule organizing center	−10.34	6.28 × 10^−23^
GO:0005654	nucleoplasm	−10.10	7.60 × 10^−22^
GO:0003677	DNA binding	−9.86	8.37 × 10^−21^
GO:0051276	chromosome organization	−9.43	5.73 × 10^−19^
GO:0007067	mitosis	−9.02	2.46 × 10^−17^
GO:0006397	mRNA processing	−8.64	7.57 × 10^−16^
GO:0009058	biosynthetic process	−8.58	1.30 × 10^−15^
GO:0005615	extracellular space	8.55	1.69 × 10^−15^
GO:0051301	cell division	−8.47	3.20 × 10^−15^
GO:0007059	chromosome segregation	−8.41	5.28 × 10^−15^
GO:0005739	mitochondrion	−8.38	7.31 × 10^−15^
GO:0043234	protein complex	−8.31	1.27 × 10^−14^
GO:0003723	RNA binding	−8.06	9.98 × 10^−14^
GO:0005929	cilium	−7.39	2.03 × 10^−11^
GO:0002376	immune system process	6.56	7.42 × 10^−09^
GO:0022607	cellular component assembly	−6.40	2.13 × 10^−08^
GO:0007267	cell-cell signaling	6.36	2.68 × 10^−08^
GO:0065003	macromolecular complex assembly	−6.19	8.19 × 10^−08^
GO:0000228	nuclear chromosome	−6.18	8.59 × 10^−08^
GO:0022857	transmembrane transporter activity	6.16	9.95 × 10^−08^
GO:0030198	extracellular matrix organization	6.08	1.63 × 10^−07^
GO:0005840	ribosome	−6.04	2.06 × 10^−07^
GO:0005829	cytosol	−5.66	2.05 × 10^−06^
GO:0007155	cell adhesion	5.56	3.53 × 10^−06^
GO:0040011	locomotion	5.44	7.15 × 10^−06^
GO:0007165	signal transduction	5.42	7.77 × 10^−06^
GO:0003735	structural constituent of ribosome	−5.42	8.01 × 10^−06^
GO:0016874	ligase activity	−5.38	1.00 × 10^−05^
GO:0050877	neurological system process	5.35	1.20 × 10^−05^
GO:0006412	translation	−5.18	2.99 × 10^−05^
GO:0005856	cytoskeleton	−5.12	4.01 × 10^−05^
GO:0043167	ion binding	−4.99	8.17 × 10^−05^
GO:0005730	nucleolus	−4.94	1.06 × 10^−04^
GO:0008168	methyltransferase activity	−4.68	3.90 × 10^−04^
GO:0048870	cell motility	4.34	0.0019
GO:0042393	histone binding	−4.15	0.0045
GO:0005578	proteinaceous extracellular matrix	4.09	0.0057
GO:0003729	mRNA binding	−4.03	0.0075
GO:0000003	reproduction	−4.01	0.0081
GO:0042592	homeostatic process	3.98	0.0091
