# Supplementary material for: An endometrial gene expression signature accurately predicts recurrent implantation failure after IVF
Source: Sci Rep. 2016 Jan 22;6:19411. doi: 10.1038/srep19411 (PMC4726345; doi:10.1038/srep19411)
Supplement: Supplementary Information [file srep19411-s1.pdf]

Supplementary Information

An endometrial gene expression signature accurately predicts recurrent implantation failure after IVF.

Yvonne E.M. Koot[1,6], Sander R. van Hooff[2,6], Carolien M. Boomsma[1], Dik van Leenen[2], Marian J.A. Groot Koerkamp[2], Mariëtte Goddijn[3], Marinus J.C. Eijkemans[1,4], Bart C.J.M. Fauser[1], Frank C.P. Holstege[2,7,*], Nick S. Macklon[1,5,7,*]

[1]Department of Reproductive Medicine and Gynaecology, University Medical Center Utrecht, Utrecht, The Netherlands. [2]Molecular Cancer Research, University Medical Center Utrecht, Utrecht, The Netherlands. [3]Center for Reproductive Medicine, Academic Medical Center, University of Amsterdam, Amsterdam, The Netherlands. [4]Julius Center for Health Sciences and Primary Care, University Medical Center Utrecht, Utrecht, The Netherlands. [5]Human Development and Health, Faculty of Medicine, University of Southampton, Southampton, United Kingdom. [6]These authors contributed equally to this work. [7]These authors contributed equally to this work.

Corresponding authors:

Nick Macklon, Email: n.s.macklon@soton.ac.uk

Frank Holstege, Email: f.c.p.holstege@umcutrecht.nl

Supplementary Figure 1

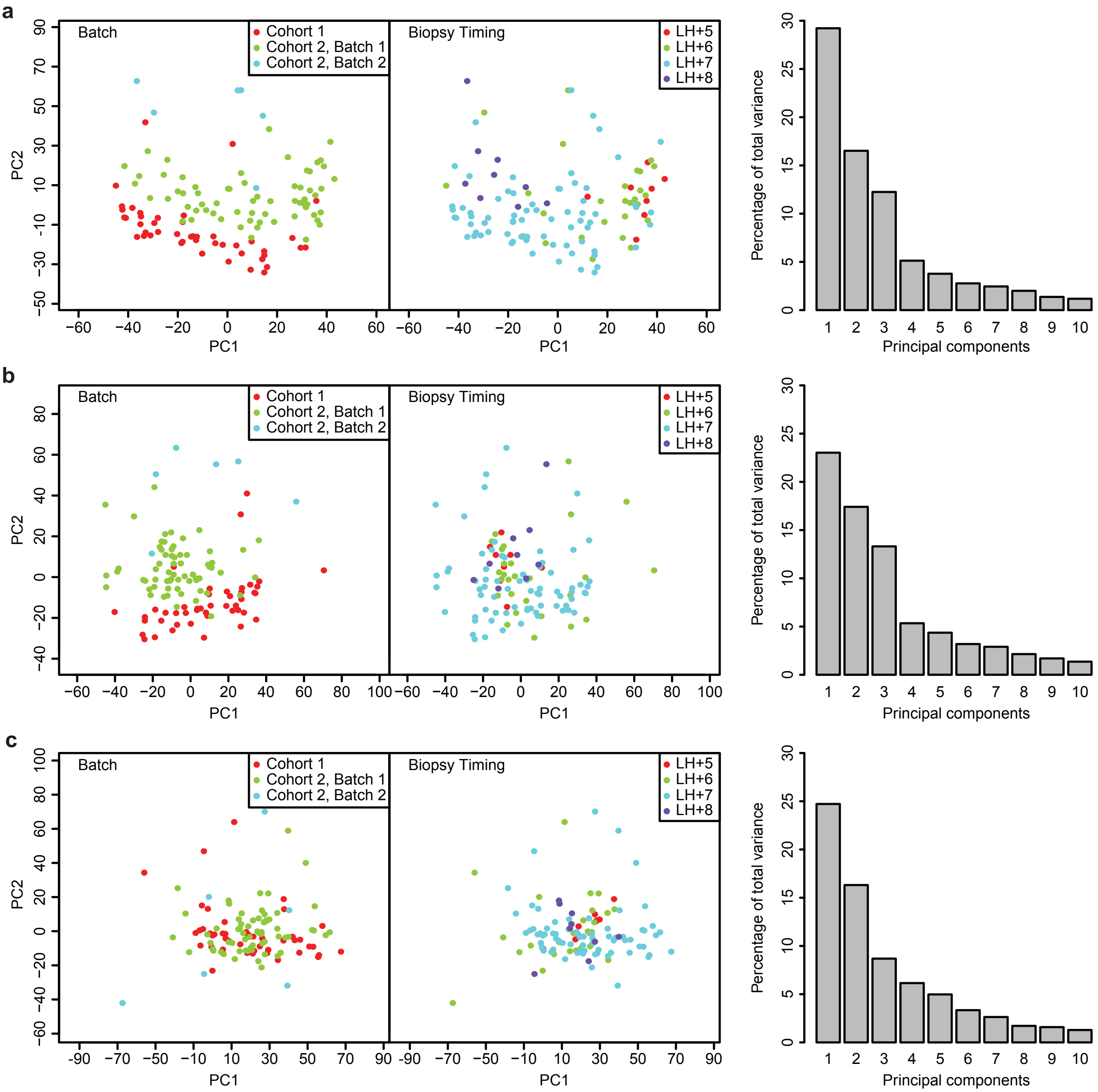

Supplementary Figure 1. Correction of biopsy timing and batch effect. A shows PCA plots for the uncorrected profiles of all samples (n=115) with batches (left) and biopsy timing (middle) superimposed. The right plot shows the amount of variation in the data explained by each of the first ten principal components. B shows the same plots but after correcting for the biopsy timing effect. C shows the results after also correcting for the batch effect.

Supplementary Figure 2

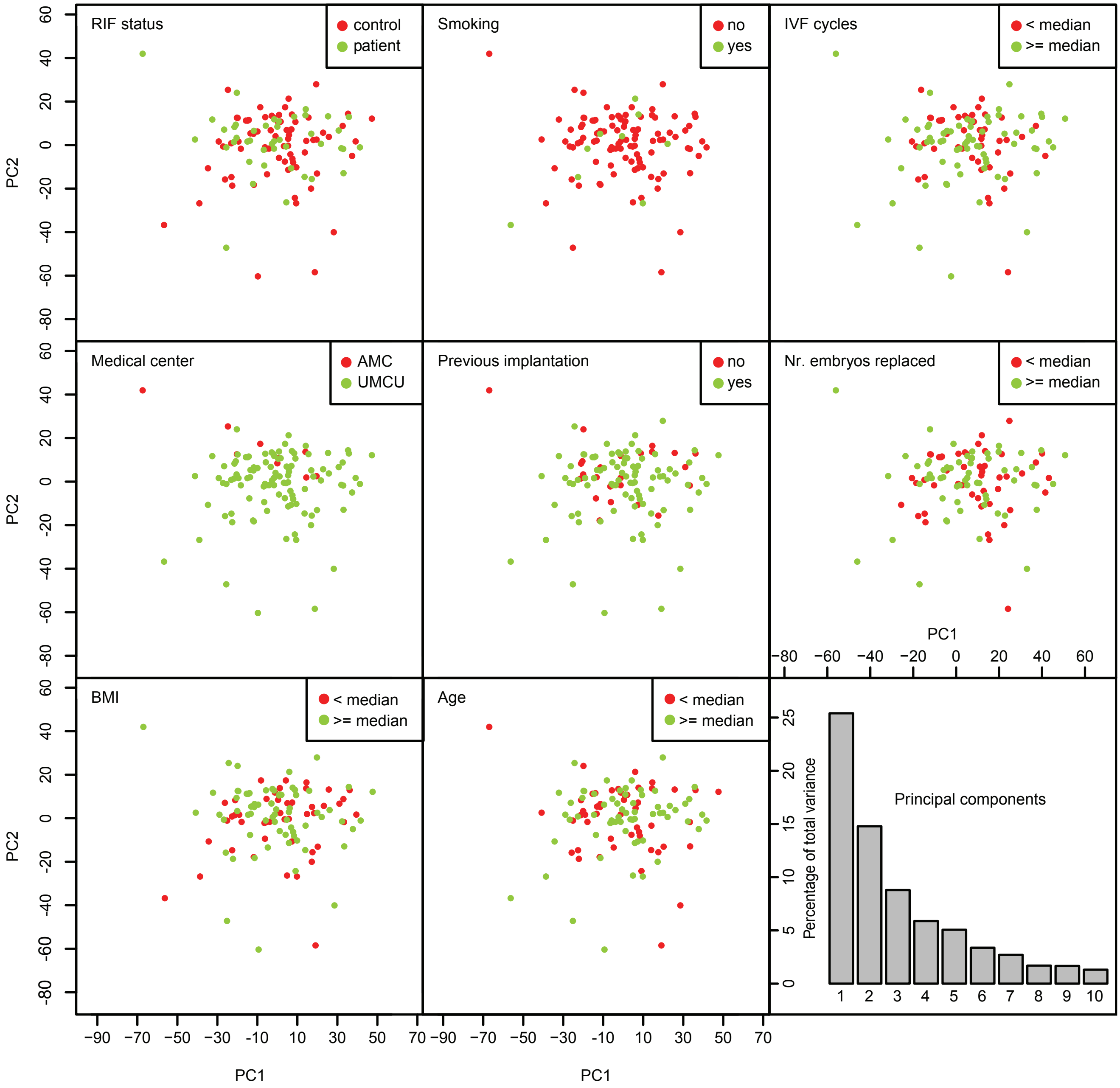

Supplementary Figure 2. PCA plots with patient characteristics and treatment variables. This figure shows PCA plots with 8 different patient characteristics and treatment variables superimposed. Continuous variables where dichotomized into below and above/equal to median value. The lower right plot shows the amount of variation in the data that is explained by each of the first ten principal components.

Supplementary Figure 3

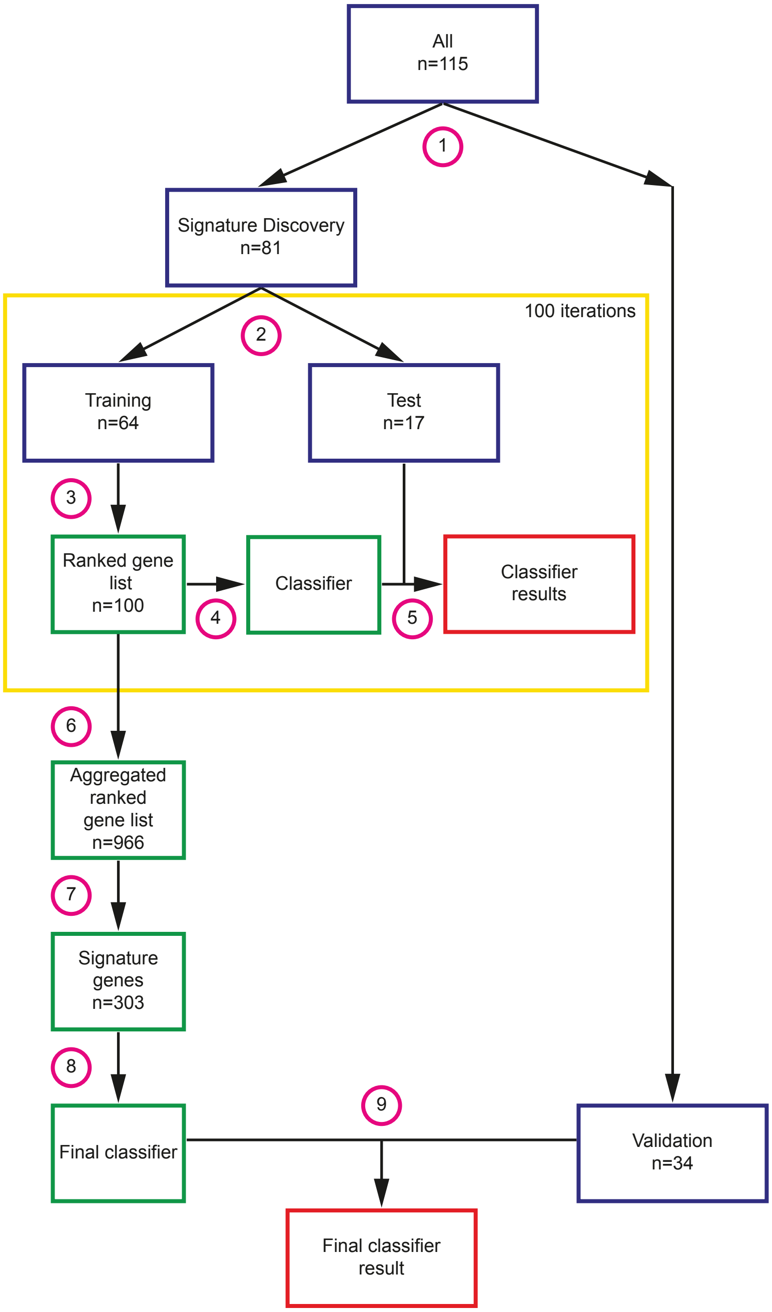

Supplementary Figure 3. Signature discovery algorithm. Diagram depicting the data used (blue boxes), analyses performed (numbered circles), output generated (green boxes) and performance measured (red boxes) during signature discovery and validation. In the first step the data is split between a signature discovery set and a validation set, step 2 involves splitting the signature discovery set into a training and test set. The training data is used to identify the 100 genes that best distinguish between RIF patient and controls (step 3). These 100 genes are employed to build a SVM classifier (step 4) which then attempts to predict the class of the samples in the test set (step 5). Step 2 to 5 are repeated 100 times which results in 100 separate 100-gene lists. These 100 lists are combined in a single list which is ranked by the occurrence of individual genes in the 100 lists (step 6). All genes which occur 10 times or more are included in the final signature (step 7) which is used to build a SVM classifier (step 8). The ninth and last step is to use the final classifier to classify the samples in the validation set.

Supplementary Figure 4

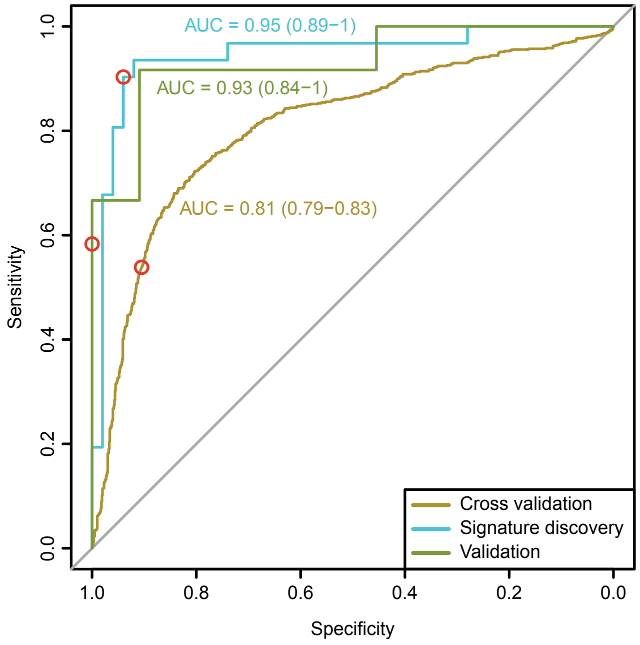

Supplementary Figure 4. Classifier ROC curves. This figure shows ROC curves for classifier performance. It is similar to Fig. 1C (blue and green curves) with an extra curve added (yellow) for the results of the 100 iterations of the cross validation procedure during signature discovery (step 5 in Supplementary Fig. S3). The Area Under the Curve (AUC) with the 95% CI is shown next to the curves. The dots indicate the point of the ROC curve that corresponds with the actual threshold used for classification (0.5).

Supplementary Figure 5

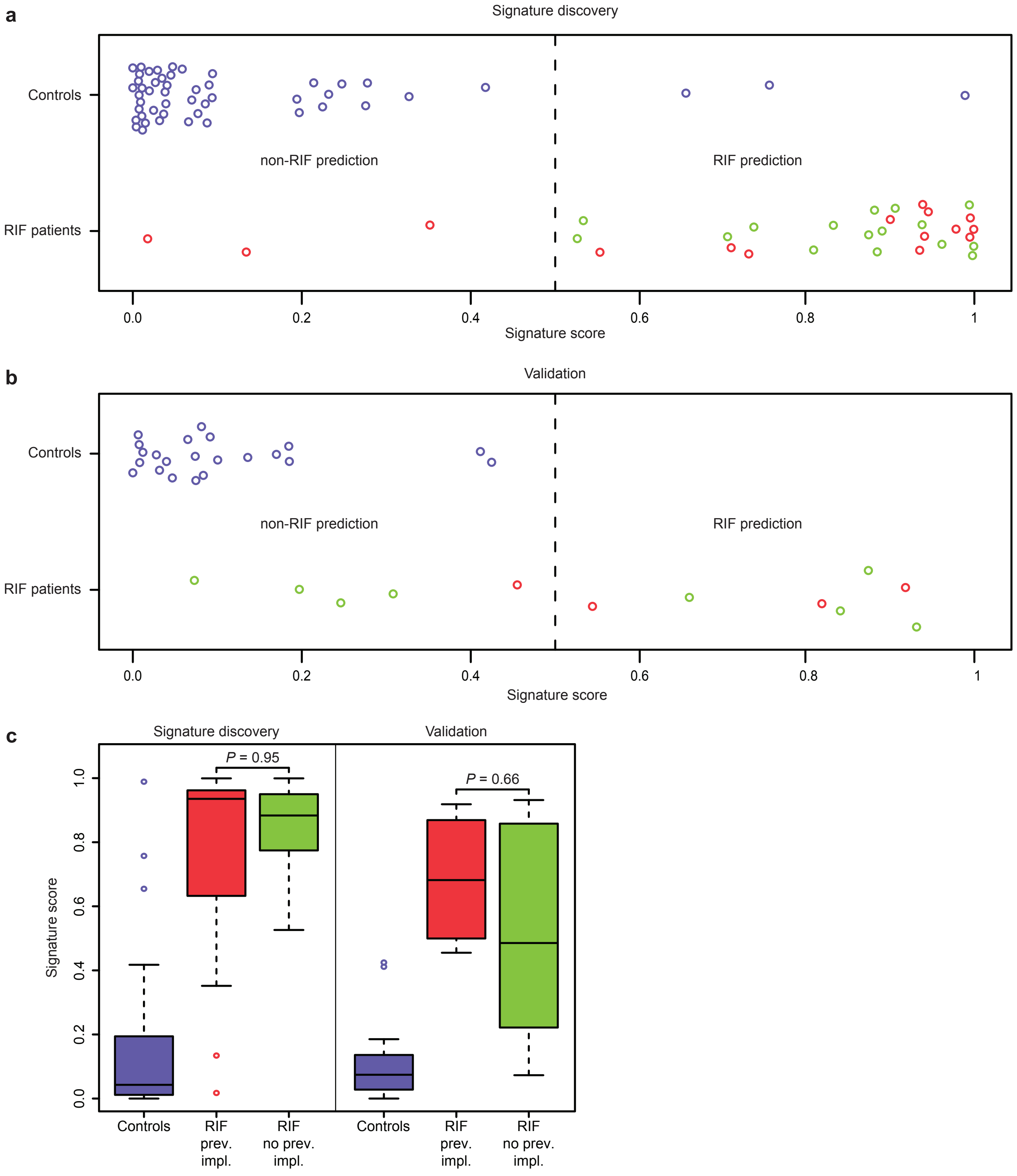

Supplementary Figure 5. Impact of previous implantation events on classifier outcome. This figure is similar to Figs. 1A and B, additionally dividing RIF patients into those with an implantation event prior to the endometrial biopsy and those without. A shows the signature scores for all RIF patients with previous implantation (red) and without previous implantation (green) and controls (blue). The signature scores are based on a classifier trained on all samples in the signature discovery set with the exception of the sample that is predicted (leave one out cross validation). All samples with a signature score below 0.5 are predicted to be controls, those with a signature score of 0.5 or higher are predicted to be RIF patients (the threshold is shown as a dotted line). B is similar to A. The samples shown are from the validation set which were scored based on a classifier trained on all samples in the signature discovery set. C shows boxplots of the signature scores of three groups; controls (blue), RIF patients with (red) and without previous implantation (green). *P* was calculated with a two-sided Mann-Whitney test.

Supplementary Figure 6

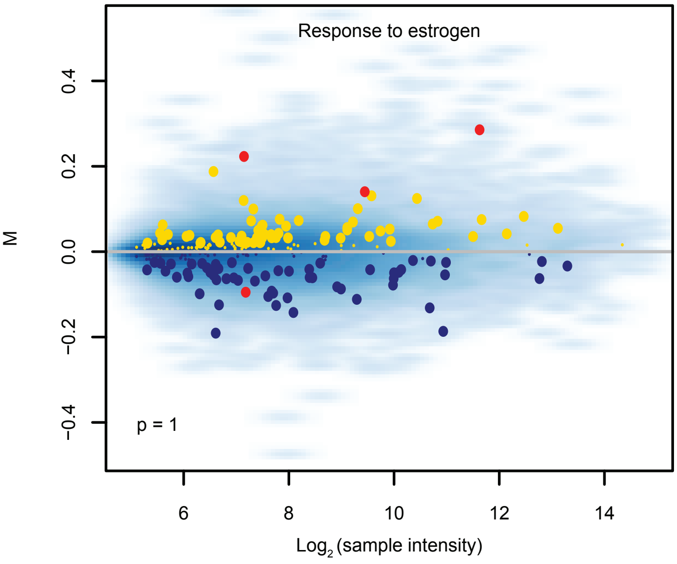

Supplementary Figure 6. Estrogen response enrichment analysis. This figure shows the gene expression of RIF patients compared against controls (log2 fold change or M) and the average expression across all samples (log2 sample intensity). Genes in the GO term 'response to estrogen' (GO:0043627) which are up-regulated in RIF patients are shown in yellow, genes down-regulated in blue. Shown in red are the genes which are also part of the 303-gene signature. Genes that are not part of the GO term are shown as a blue density map where darker blue indicates higher gene density.

Supplementary Figure 7

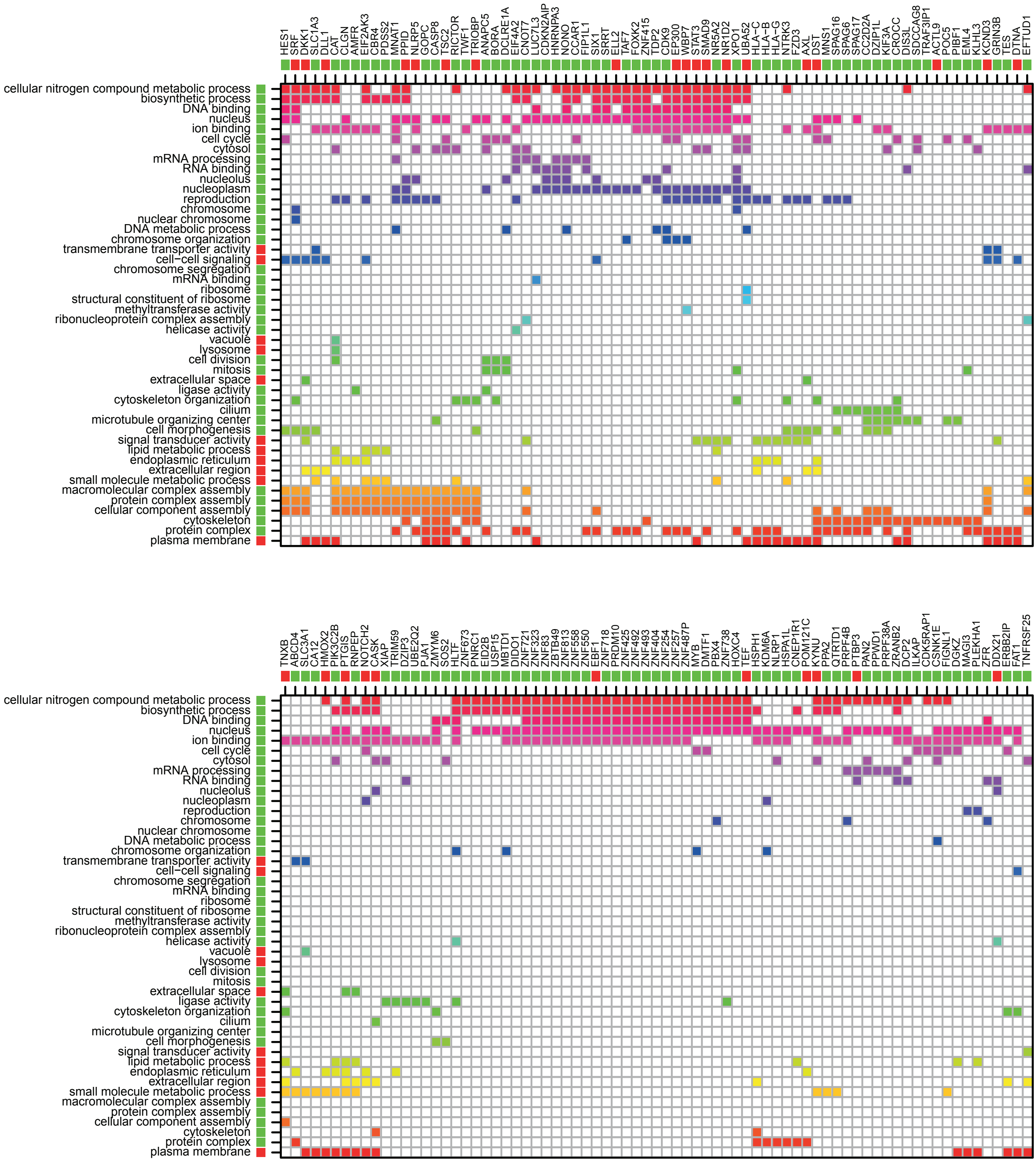

Supplementary Figure 7. Functional characterisation 303-gene signature. This figure is similar to Fig. 2E but now showing all signature genes (n=296) present in the GO database.

Supplementary Figure 7 continued

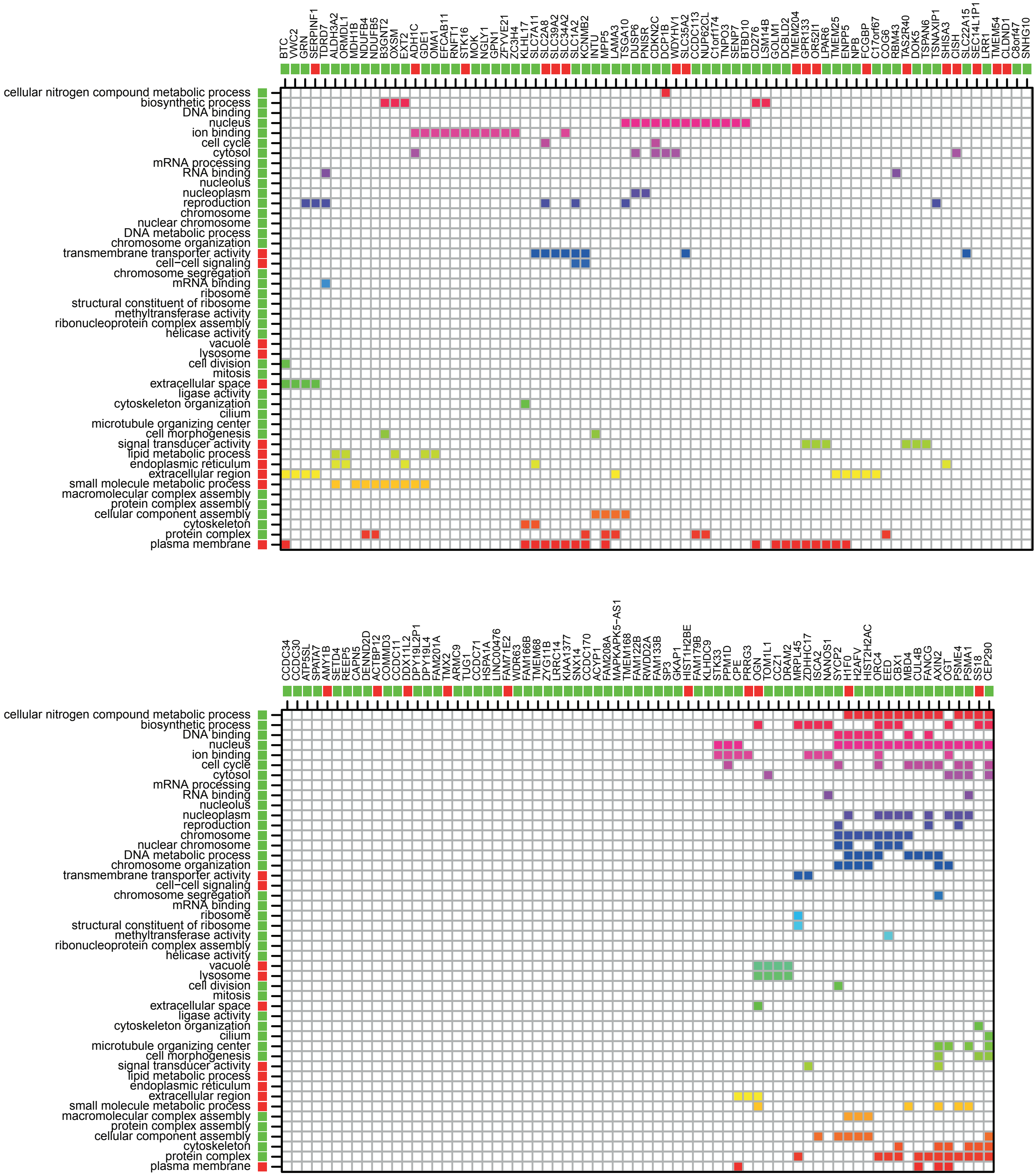

TableS1. PCA of biopsy timing and batch effect corrections

	P			
	**PC1**	**PC2**	**PC3**	**PC4**
**(A) Before correction**				
**Biopsy timing**				
**LH+5**	4.2×10^{-5}	0.178	0.382	0.047
**LH+6**	2.0×10^{-5}	0.157	0.623	0.490
**LH+7**	4.2×10^{-5}	3.4×10^{-4}	0.058	0.016
**LH+8**	0.003	0.003	0.069	0.171
**Batch**				
**Cohort 1**	4.2×10^{-5}	9.7×10^{-12}	1.3×10^{-7}	0.866
**Cohort 2, Batch 1**	2.0×10^{-5}	6.2×10^{-7}	2.0×10^{-8}	0.403
**Cohort 2, Batch 2**	0.593	1.4×10^{-4}	0.342	0.136
**(B) After correction for biopsy timing**				
**Biopsy timing**				
**LH+5**	0.320	0.072	0.779	0.342
**LH+6**	0.091	0.591	0.546	0.825
**LH+7**	0.432	0.006	0.110	0.399
**LH+8**	0.767	0.016	0.097	0.330
**Batch**				
**Cohort 1**	3.0×10^{-4}	1.4×10^{-12}	5.1×10^{-7}	0.306
**Cohort 2, Batch 1**	1.6×10^{-4}	1.7×10^{-7}	1.0×10^{-7}	0.086
**Cohort 2, Batch 2**	0.628	1.2×10^{-4}	0.389	0.114
**(C) After correction for batch**				
**Biopsy timing**				
**LH+5**	0.407	0.081	0.438	0.520
**LH+6**	0.079	0.815	0.002	0.699
**LH+7**	0.137	0.158	0.098	0.263
**LH+8**	0.482	0.599	0.286	0.426
**Batch**				
**Cohort 1**	0.580	0.175	0.938	0.588
**Cohort 2, Batch 1**	0.426	0.144	0.763	0.575
**Cohort 2, Batch 2**	0.576	0.777	0.619	0.955

P was calculated using a two-sided Mann-Whitney test contrasting the PC values of samples with a particular variable value against the rest (i.e. PC1 values of LH+5 vs. LH+6/7/8); PC1-4, principal component 1-4; LH+X, biopsy was taken X days after luteinizing hormone (LH) surge.

TableS2. Variable association with principal components

	P			
**Variables**	**PC1**	**PC2**	**PC3**	**PC4**
**RIF status (patient/control)**	0.577	0.692	0.561	0.209
**Medical center (UMCU/AMC)**	0.556	0.002	0.606	0.979
**BMI (median)**	0.349	0.315	0.507	0.157
**Smoking (yes/no)**	0.571	0.549	0.760	0.744
**Implantations (median)**	0.618	0.183	0.855	0.910
**Age (median)**	0.768	0.913	0.750	0.953
**IVF cycles (median)**	0.845	0.233	0.468	0.292
**Nr. embryos replaced (median)**	0.430	0.791	0.949	0.313

P was calculated using a two-sided Mann-Whitney test contrasting the PC values of one group against the other group (i.e. PC1 values of smokers vs. non-smokers); PC1-4, principal component 1-4; Median, the median value was used to dichotomize the covariates (< median & ≥ median); BMI, body mass index.

Table S3. Signature genes

Probe ID	Gene symbol	Description	M
**HSAE000532**	PPWD1	peptidylprolyl isomerase domain and WD repeat containing 1	-0.19
**HSAE022203**	POC5	POC5 centriolar protein homolog (Chlamydomonas)	-0.17
**HSAE022471**	CSNK1E	casein kinase 1, epsilon	-0.12
**HSAE019770**	FAM179B	family with sequence similarity 179, member B	-0.19
**HSAE005427**	CPE	carboxypeptidase E	-0.48
**HSAE006018**	DCP1B	DCP1 decapping enzyme homolog B (S. cerevisiae)	-0.11
**HSAE010975**	TAF7	TAF7 RNA polymerase II, TATA box binding protein (TBP)-associated factor, 55kDa	-0.15
**HSAE014616**	SLC1A2	solute carrier family 1 (glial high affinity glutamate transporter), member 2	-0.24
**HSAE021377**	MPP5	membrane protein, palmitoylated 5 (MAGUK p55 subfamily member 5)	-0.11
**HSAE021192**	OXSM	3-oxoacyl-ACP synthase, mitochondrial	-0.12
**HSAE008217**	EP300	E1A binding protein p300	0.19
**HSAE030183**	TNXB	tenascin XB	0.12
**HSAE011196**	KLHDC9	kelch domain containing 9	-0.15
**HSAE007316**	EID2B	EP300 interacting inhibitor of differentiation 2B	-0.10
**HSAE000275**	CAT	Catalase	-0.17
**HSAE020379**	CBR4	carbonyl reductase 4	-0.16
**HSAE003961**	TMEM25	transmembrane protein 25	-0.13
**HSAE007811**	SLC22A15	solute carrier family 22, member 15	-0.11
**HSAE002494**	CUL4B	cullin 4B	-0.12
**HSAE004566**	FCGBP	Fc fragment of IgG binding protein	0.14
**HSAE012530**	CASP8	caspase 8, apoptosis-related cysteine peptidase	-0.07
**HSAE001942**	OMA1	OMA1 zinc metallopeptidase homolog (S. cerevisiae)	-0.11
**HSAE014904**	CCZ1	CCZ1 vacuolar protein trafficking and biogenesis associated homolog (S. cerevisiae)	-0.05
**HSAE031372**	ZNF257	zinc finger protein 257	-0.13
**HSAE002376**	H2AFV	H2A histone family, member V	-0.14
**HSAE016014**	CCDC113	coiled-coil domain containing 113	-0.20
**HSAE032669**	ZNF415	zinc finger protein 415	-0.18
**HSAE013562**	ZMYM6	zinc finger, MYM-type 6	-0.12
**HSAE015519**	RP1-152L7.5	RP1-152L7.5	-0.11
**HSAE002271**	DZIP3	DAZ interacting protein 3, zinc finger	-0.17
**HSAE004693**	ZNF673	zinc finger family member 673	-0.13
**HSAE011062**	TDP2	tyrosyl-DNA phosphodiesterase 2	-0.12
**HSAE033287**	ZNF487P	zinc finger protein 487, pseudogene	-0.15
**HSAE020170**	ZNF738	zinc finger protein 738	-0.19
**HSAE031067**	KCNMB2	potassium large conductance calcium-activated channel, subfamily M, beta member 2	-0.19
**HSAE011386**	LAMA3	laminin, alpha 3	-0.25
**HSAE026892**	NLRP1	NLR family, pyrin domain containing 1	-0.23
**HSAE007958**	DIDO1	death inducer-obliterator 1	-0.10
**HSAE018217**	RP5-1129J21.3	RP5-1129J21.3	-0.18
**HSAE002930**	TNXB	tenascin XB	0.11
**HSAE023427**	CTD-2116N17.1	CTD-2116N17.1	0.19
**HSAE024971**	HIST1H2BE	histone cluster 1, H2be	0.09
**HSAE022135**	AC007292.6	AC007292.6	0.12
**HSAE011646**	GKAP1	G kinase anchoring protein 1	-0.17
**HSAE006812**	SP3	Sp3 transcription factor	-0.20
**HSAE007705**	DZIP1L	DAZ interacting protein 1-like	-0.21
**HSAE000804**	CCAR1	cell division cycle and apoptosis regulator 1	-0.10
**HSAE016921**	INTU	inturned planar cell polarity effector homolog (Drosophila)	-0.15
**HSAE012539**	FAM133B	family with sequence similarity 133, member B	-0.10
**HSAE013456**	ZRANB2	zinc finger, RAN-binding domain containing 2	-0.17
**HSAE002193**	ZFYVE21	zinc finger, FYVE domain containing 21	-0.10
**HSAE021606**	RP11-706O15.3	RP11-706O15.3	-0.07

**HSAE019450**	ZNF254	zinc finger protein 254	-0.14
**HSAE030937**	FOXK2	forkhead box K2	-0.06
**HSAE002268**	CISH	cytokine inducible SH2-containing protein	0.22
**HSAE012322**	DCBLD2	discoidin, CUB and LCCL domain containing 2	-0.20
**HSAE000881**	SOS2	son of sevenless homolog 2 (Drosophila)	-0.10
**HSAE001077**	EIF4A2	eukaryotic translation initiation factor 4A2	-0.12
**HSAE009803**	ELL2	elongation factor, RNA polymerase II, 2	0.07
**HSAE034109**	PPA2	pyrophosphatase (inorganic) 2	-0.12
**HSAE009179**	TWF1	twinfilin, actin-binding protein, homolog 1 (Drosophila)	-0.10
**HSAE009528**	EED	embryonic ectoderm development	-0.20
**HSAE005215**	RWDD2A	RWD domain containing 2A	-0.08
**HSAE013680**	SENP7	SUMO1/sentrin specific peptidase 7	-0.23
**HSAE001761**	BTBD10	BTB (POZ) domain containing 10	-0.06
**HSAE032952**	TNPO3	transportin 3	-0.03
**HSAE012052**	KYNU	kynureninase	0.52
**HSAE014369**	CA12	carbonic anhydrase XII	-0.47
**HSAE008008**	CNEP1R1	CTD nuclear envelope phosphatase 1 regulatory subunit 1	-0.03
**HSAE010272**	SPAG16	sperm associated antigen 16	-0.11
**HSAE015672**	C17orf67	chromosome 17 open reading frame 67	-0.09
**HSAE033077**	KIF3A	kinesin family member 3A	-0.09
**HSAE011415**	ZNF404	zinc finger protein 404	-0.29
**HSAE013758**	EML4	echinoderm microtubule associated protein like 4	-0.11
**HSAE023428**	OGN	osteoglycin	0.21
**HSAE008504**	ZFR	zinc finger RNA binding protein	-0.10
**HSAE010484**	FANCG	Fanconi anemia, complementation group G	-0.11
**HSAE027480**	KDM6A	lysine (K)-specific demethylase 6A	-0.09
**HSAE016847**	ZNF493	zinc finger protein 493	-0.13
**HSAE001943**	OMA1	OMA1 zinc metallopeptidase homolog (S. cerevisiae)	-0.06
**HSAE022240**	SLC35A2	solute carrier family 35 (UDP-galactose transporter), member A2	0.14
**HSAE005948**	MYB	v-myb myeloblastosis viral oncogene homolog (avian)	-0.26
**HSAE018195**	KCND3	potassium voltage-gated channel, Shal-related subfamily, member 3	0.09
**HSAE018201**	MBTD1	mbt domain containing 1	-0.12
**HSAE002138**	SS18	synovial sarcoma translocation, chromosome 18	0.03
**HSAE027412**	DST	dystonin	0.06
**HSAE015476**	PTGIS	prostaglandin I2 (prostacyclin) synthase	0.19
**HSAE011055**	OGN	osteoglycin	0.19
**HSAE020336**	FAM122B	family with sequence similarity 122B	-0.22
**HSAE015317**	TMEM168	transmembrane protein 168	-0.14
**HSAE004016**	USP15	ubiquitin specific peptidase 15	-0.16
**HSAE006357**	MNAT1	menage a trois homolog 1, cyclin H assembly factor (Xenopus laevis)	-0.10
**HSAE000600**	TES	testis derived transcript (3 LIM domains)	-0.03
**HSAE000934**	TOM1L1	target of myb1 (chicken)-like 1	-0.05
**HSAE011421**	PBX4	pre-B-cell leukemia homeobox 4	-0.08
**HSAE014003**	CBX1	chromobox homolog 1	-0.14
**HSAE001338**	TNFRSF25	tumor necrosis factor receptor superfamily, member 25	-0.16
**HSAE014032**	C1orf174	chromosome 1 open reading frame 174	-0.06
**HSAE019250**	MDH1B	malate dehydrogenase 1B, NAD (soluble)	-0.18
**HSAE011842**	MAPKAPK5-AS1	MAPKAPK5 antisense RNA 1	-0.06
**HSAE001836**	EIF2AK3	eukaryotic translation initiation factor 2-alpha kinase 3	-0.09
**HSAE013221**	ZC3H4	zinc finger CCCH-type containing 4	-0.10
**HSAE010205**	FAM208A	family with sequence similarity 208, member A	-0.08
**HSAE021043**	PSME4	proteasome (prosome, macropain) activator subunit 4	-0.14
**HSAE013883**	SLC1A3	solute carrier family 1 (glial high affinity glutamate transporter), member 3	-0.14
**HSAE002020**	DUSP6	dual specificity phosphatase 6	-0.19
**HSAE003248**	ACYP1	acylphosphatase 1, erythrocyte (common) type	-0.12
**HSAE011498**	QTRTD1	queuine tRNA-ribosyltransferase domain containing 1	-0.08
**HSAE018659**	ETV6	ets variant 6	-0.10

**HSAE001585**	ORC4	origin recognition complex, subunit 4	-0.10
**HSAE022833**	CCDC170	coiled-coil domain containing 170	-0.25
**HSAE003644**	GPN1	GPN-loop GTPase 1	-0.08
**HSAE004788**	NGLY1	N-glycanase 1	-0.11
**HSAE010725**	MOK	MOK protein kinase	-0.11
**HSAE005403**	SNX14	sorting nexin 14	-0.13
**HSAE004343**	SYCP2	synaptonemal complex protein 2	-0.15
**HSAE002779**	ZNF492	zinc finger protein 492	-0.10
**HSAE018198**	KIAA1377	KIAA1377	-0.15
**HSAE002663**	ISCA2	iron-sulfur cluster assembly 2 homolog (S. cerevisiae)	-0.07
**HSAE029538**	DTNA	dystrobrevin, alpha	0.15
**HSAE015424**	LRRC10B	leucine rich repeat containing 10B	-0.18
**HSAE014744**	LRRC14	leucine rich repeat containing 14	-0.14
**HSAE005719**	ERBB2IP	erbb2 interacting protein	-0.12
**HSAE008692**	ZYG11B	zyg-11 homolog B (C. elegans)	-0.10
**HSAE020721**	DGKZ	diacylglycerol kinase, zeta	-0.09
**HSAE016618**	ZNF425	zinc finger protein 425	-0.05
**HSAE001849**	TMEM68	transmembrane protein 68	-0.05
**HSAE016437**	RP11-686D22.8	RP11-686D22.8	-0.11
**HSAE004102**	FAM166B	family with sequence similarity 166, member B	-0.07
**HSAE001830**	BTC	betacellulin	-0.06
**HSAE008612**	WDR63	WD repeat domain 63	-0.12
**HSAE014596**	FAM71E2	family with sequence similarity 71, member E2	0.12
**HSAE000694**	MRPL45	mitochondrial ribosomal protein L45	-0.05
**HSAE019606**	LINC00476	long intergenic non-protein coding RNA 476	-0.11
**HSAE001712**	KLHL3	kelch-like 3 (Drosophila)	-0.15
**HSAE000571**	HSPA1A	heat shock 70kDa protein 1A	-0.16
**HSAE009957**	CCDC71	coiled-coil domain containing 71	-0.04
**HSAE024874**	HOXC4	homeobox C4	-0.13
**HSAE007783**	PRRG3	proline rich Gla (G-carboxyglutamic acid) 3 (transmembrane)	0.15
**HSAE003836**	PIK3C2B	phosphatidylinositol-4-phosphate 3-kinase, catalytic subunit type 2 beta	-0.17
**HSAE017699**	PRDM10	PR domain containing 10	-0.12
**HSAE033212**	Unknown	Unknown	-0.22
**HSAE029507**	TUG1	taurine upregulated 1 (non-protein coding)	-0.16
**HSAE005634**	H1F0	H1 histone family, member 0	0.32
**HSAE018292**	TRIM59	tripartite motif containing 59	-0.21
**HSAE004297**	ARMC9	armadillo repeat containing 9	-0.17
**HSAE029390**	MAGI3	membrane associated guanylate kinase, WW and PDZ domain containing 3	-0.13
**HSAE002178**	WBP7	Histone-lysine N-methyltransferase MLL4	0.07
**HSAE018961**	PDSS2	prenyl (decaprenyl) diphosphate synthase, subunit 2	-0.03
**HSAE010367**	NDUFB4	NADH dehydrogenase (ubiquinone) 1 beta subcomplex, 4, 15kDa	-0.05
**HSAE001227**	ZNF718	zinc finger protein 718	-0.06
**HSAE012481**	TMX2	thioredoxin-related transmembrane protein 2	0.07
**HSAE013709**	PLEKHA1	pleckstrin homology domain containing, family A (phosphoinositide binding specific) member 1	-0.08
**HSAE001201**	TDRD7	tudor domain containing 7	-0.06
**HSAE005355**	CDKN2AIP	CDKN2A interacting protein	-0.14
**HSAE032816**	KLHL17	kelch-like 17 (Drosophila)	-0.10
**HSAE012522**	EBF1	early B-cell factor 1	0.04
**HSAE022046**	NOTCH2	notch 2	0.12
**HSAE030479**	OR52I1	olfactory receptor, family 52, subfamily I, member 1	0.08
**HSAE010170**	DIS3L	DIS3 mitotic control homolog (S. cerevisiae)-like	-0.05
**HSAE019411**	RP11-689P11.2	RP11-689P11.2	-0.12
**HSAE030182**	FAM201A	family with sequence similarity 201, member A	-0.07
**HSAE017450**	DPY19L4	dpy-19-like 4 (C. elegans)	-0.12
**HSAE021251**	XIAP	X-linked inhibitor of apoptosis	-0.11
**HSAE027237**	ALDH3A2	aldehyde dehydrogenase 3 family, member A2	-0.17

**HSAE012753**	DPY19L2P1	dpy-19-like 2 pseudogene 1 (C. elegans)	-0.13
**HSAE001450**	SLC39A2	solute carrier family 39 (zinc transporter), member 2	0.06
**HSAE017691**	DDX11L2	DEAD/H (Asp-Glu-Ala-Asp/His) box polypeptide 11 like 2	0.17
**HSAE010102**	PAN2	PAN2 poly(A) specific ribonuclease subunit homolog (S. cerevisiae)	-0.12
**HSAE030728**	NR5A2	nuclear receptor subfamily 5, group A, member 2	-0.13
**HSAE007702**	CCDC11	coiled-coil domain containing 11	-0.12
**HSAE024253**	LUC7L3	LUC7-like 3 (S. cerevisiae)	-0.21
**HSAE010180**	HES1	hairy and enhancer of split 1, (Drosophila)	-0.13
**HSAE003618**	TSGA10	testis specific, 10	-0.13
**HSAE014580**	POM121C	POM121 transmembrane nucleoporin C	0.02
**HSAE006626**	NUP62CL	nucleoporin 62kDa C-terminal like	-0.16
**HSAE001620**	EXT2	exostosin 2	-0.13
**HSAE009603**	GDE1	glycerophosphodiester phosphodiesterase 1	-0.08
**HSAE024589**	NDUFB5	NADH dehydrogenase (ubiquinone) 1 beta subcomplex, 5, 16kDa	-0.12
**HSAE010188**	TSC2	tuberous sclerosis 2	0.13
**HSAE016743**	PRPF38A	PRP38 pre-mRNA processing factor 38 (yeast) domain containing A	-0.10
**HSAE032880**	ZNF730	Zinc finger protein 730	-0.21
**HSAE009304**	B3GNT2	UDP-GlcNAc:betaGal beta-1,3-N-acetylglucosaminyltransferase 2	-0.04
**HSAE005110**	SLC2A8	solute carrier family 2 (facilitated glucose transporter), member 8	0.10
**HSAE023646**	TMEM204	transmembrane protein 204	0.12
**HSAE008410**	SHISA3	shisa homolog 3 (Xenopus laevis)	0.23
**HSAE017269**	TRAF3IP1	TNF receptor-associated factor 3 interacting protein 1	-0.08
**HSAE006817**	FZD3	frizzled family receptor 3	-0.18
**HSAE001956**	COMMD3	COMM domain containing 3	-0.10
**HSAE005511**	LPAR6	lysophosphatidic acid receptor 6	-0.16
**HSAE019557**	CD276	CD276 molecule	-0.08
**HSAE011219**	DMTF1	cyclin D binding myb-like transcription factor 1	-0.14
**HSAE019844**	OGT	O-linked N-acetylglucosamine (GlcNAc) transferase	-0.21
**HSAE007934**	CNOT7	CCR4-NOT transcription complex, subunit 7	-0.11
**HSAE020945**	SLC7A11	solute carrier family 7 (anionic amino acid transporter light chain, xc- system), member 11	-0.21
**HSAE030082**	HLA-B	major histocompatibility complex, class I, B	-0.27
**HSAE017421**	Unknown	Unknown	-0.07
**HSAE025366**	CC2D2A	coiled-coil and C2 domain containing 2A	-0.18
**HSAE003728**	CLGN	calmegin	-0.21
**HSAE014978**	NANOS1	nanos homolog 1 (Drosophila)	-0.07
**HSAE024968**	GPR133	G protein-coupled receptor 133	0.08
**HSAE001491**	TSNAXIP1	translin-associated factor X interacting protein 1	-0.14
**HSAE010427**	HSPA1L	heat shock 70kDa protein 1-like	-0.06
**HSAE023884**	GRN	granulin	-0.06
**HSAE000025**	HMOX2	heme oxygenase (decycling) 2	0.08
**HSAE005268**	BORA	bora, aurora kinase A activator	-0.14
**HSAE008114**	DOK5	docking protein 5	-0.17
**HSAE013693**	DRAM2	DNA-damage regulated autophagy modulator 2	-0.08
**HSAE001682**	SLC34A2	solute carrier family 34 (sodium phosphate), member 2	0.28
**HSAE033332**	Unknown	Unknown	0.09
**HSAE012471**	NONO	non-POU domain containing, octamer-binding	-0.09
**HSAE032360**	ACTBP12	actin, beta pseudogene 12	0.12
**HSAE004298**	ARMC9	armadillo repeat containing 9	-0.13
**HSAE011189**	DENND2D	DENN/MADD domain containing 2D	-0.10
**HSAE030948**	CAPN5	calpain 5	-0.13
**HSAE004030**	ANAPC5	anaphase promoting complex subunit 5	-0.08
**HSAE008084**	XPO1	exportin 1 (CRM1 homolog, yeast)	-0.10
**HSAE002092**	AMFR	autocrine motility factor receptor, E3 ubiquitin protein ligase	-0.33
**HSAE006303**	LSM14B	LSM14B, SCD6 homolog B (S. cerevisiae)	-0.03
**HSAE014463**	SRF	serum response factor (c-fos serum response element-binding transcription factor)	0.06
**HSAE001396**	STK33	serine/threonine kinase 33	-0.15

**HSAE017690**	FAM126B	family with sequence similarity 126, member	-0.14
**HSAE020161**	GOLM1	golgi membrane protein 1	-0.05
**HSAE002216**	EML4	echinoderm microtubule associated protein like 4	-0.18
**HSAE006880**	REEP5	receptor accessory protein 5	-0.07
**HSAE007003**	ZNF550	zinc finger protein 550	-0.10
**HSAE005704**	HIST2H2AC	histone cluster 2, H2ac	-0.17
**HSAE013304**	SETD4	SET domain containing 4	-0.09
**HSAE001016**	ORMDL1	ORM1-like 1 (S. cerevisiae)	-0.11
**HSAE012469**	TEF	thyrotrophic embryonic factor	0.03
**HSAE023950**	IQCD	IQ motif containing D	-0.05
**HSAE029097**	AMY1B	amylase, alpha 1B (salivary)	0.48
**HSAE017836**	SPATA7	spermatogenesis associated 7	-0.12
**HSAE002184**	ILKAP	integrin-linked kinase-associated serine/threonine phosphatase	-0.04
**HSAE005751**	FIGNL1	fidgetin-like 1	-0.12
**HSAE004529**	ENPP5	ectonucleotide pyrophosphatase/phosphodiesterase 5 (putative)	-0.08
**HSAE017429**	RICTOR	RPTOR independent companion of MTOR, complex 2	-0.13
**HSAE005562**	ZNF558	zinc finger protein 558	-0.11
**HSAE027741**	ZNF813	zinc finger protein 813	-0.03
**HSAE010738**	SIX1	SIX homeobox 1	-0.08
**HSAE012506**	ZBTB49	zinc finger and BTB domain containing 49	-0.10
**HSAE033552**	CROCC	ciliary rootlet coiled-coil, rootletin	-0.12
**HSAE032557**	SLC3A1	solute carrier family 3 (cystine, dibasic and neutral amino acid transporters, activator of cystine, dibasic and neutral amino acid transport), member 1	-0.06
**HSAE021990**	RNPEP	arginyl aminopeptidase (aminopeptidase B)	-0.07
**HSAE032660**	NPB	neuropeptide B	-0.08
**HSAE000414**	ATP5SL	ATP5S-like	-0.04
**HSAE014170**	HLA-G	major histocompatibility complex, class I, G	-0.18
**HSAE032969**	HLA-C	major histocompatibility complex, class I, C	-0.19
**HSAE016907**	RBM43	RNA binding motif protein 43	-0.12
**HSAE021891**	ZNF83	zinc finger protein 83	-0.14
**HSAE008338**	PIBF1	progesterone immunomodulatory binding factor 1	-0.09
**HSAE003626**	TSPAN6	tetraspanin 6	-0.18
**HSAE013158**	SPAG6	sperm associated antigen 6	-0.26
**HSAE024786**	SRRT	serrate RNA effector molecule homolog (Arabidopsis)	-0.08
**HSAE027749**	ACTL9	actin-like 9	0.07
**HSAE002971**	VWC2	von Willebrand factor C domain containing 2	-0.23
**HSAE002682**	EFTUD1	elongation factor Tu GTP binding domain containing 1	-0.06
**HSAE026502**	TRIOBP	TRIO and F-actin binding protein	0.07
**HSAE009253**	NLRP5	NLR family, pyrin domain containing 5	0.15
**HSAE000440**	STK16	serine/threonine kinase 16	0.06
**HSAE007450**	ABCD4	ATP-binding cassette, sub-family D (ALD), member 4	-0.12
**HSAE004543**	ZNF323	zinc finger protein 323	-0.15
**HSAE006633**	TAS2R40	taste receptor, type 2, member 40	0.27
**HSAE017069**	NTRK3	neurotrophic tyrosine kinase, receptor, type 3	0.22
**HSAE012776**	SDCCAG8	serologically defined colon cancer antigen 8	-0.02
**HSAE013790**	HLTF	helicase-like transcription factor	-0.19
**HSAE011512**	MBD4	methyl-CpG binding domain protein 4	-0.07
**HSAE006537**	ZNF721	zinc finger protein 721	-0.08
**HSAE014584**	CCDC30	coiled-coil domain containing 30	-0.10
**HSAE010374**	FAT1	FAT tumor suppressor homolog 1 (Drosophila)	-0.11
**HSAE024319**	CDKN2C	cyclin-dependent kinase inhibitor 2C (p18, inhibits CDK4)	-0.22
**HSAE006041**	ZDHHC17	zinc finger, DHHC-type containing 17	-0.18
**HSAE025704**	CCDC34	coiled-coil domain containing 34	-0.19
**HSAE007323**	CEP290	centrosomal protein 290kDa	-0.15
**HSAE027046**	UBE2Q2	ubiquitin-conjugating enzyme E2Q family member 2	-0.13
**HSAE012714**	UBA52	ubiquitin A-52 residue ribosomal protein fusion product 1	0.06
**HSAE003564**	CDK9	cyclin-dependent kinase 9	-0.07

**HSAE026293**	Unknown	Unknown	0.08
**HSAE010971**	ADH1C	alcohol dehydrogenase 1C (class I), gamma polypeptide	0.05
**HSAE000034**	SERPINF1	serpin peptidase inhibitor, clade F (alpha-2 antiplasmin, pigment epithelium derived factor), member 1	0.19
**HSAE031007**	GRIN3B	glutamate receptor, ionotropic, N-methyl-D-aspartate 3B	-0.14
**HSAE004172**	RNFT1	ring finger protein, transmembrane 1	-0.10
**HSAE011288**	HSPH1	heat shock 105kDa/110kDa protein 1	-0.19
**HSAE018395**	SNHG10	small nucleolar RNA host gene 10 (non-protein coding)	-0.12
**HSAE017258**	CASK	calcium/calmodulin-dependent serine protein kinase (MAGUK family)	0.04
**HSAE019166**	PJA1	praja ring finger 1, E3 ubiquitin protein ligase	-0.17
**HSAE025842**	PTBP3	polypyrimidine tract binding protein 3	0.09
**HSAE007756**	PPID	peptidylprolyl isomerase D	0.02
**HSAE006495**	C8orf47	chromosome 8 open reading frame 47	-0.14
**HSAE002475**	AXIN2	axin 2	-0.10
**HSAE006190**	SPAG17	sperm associated antigen 17	-0.23
**HSAE016684**	NR1D2	nuclear receptor subfamily 1, group D, member 2	0.12
**HSAE002873**	CLDND1	claudin domain containing 1	0.08
**HSAE016637**	PRPF4B	PRP4 pre-mRNA processing factor 4 homolog B (yeast)	-0.17
**HSAE029270**	TMEM215	Transmembrane protein 215	-0.07
**HSAE009822**	GOPC	golgi-associated PDZ and coiled-coil motif containing	-0.15
**HSAE017125**	PPM1D	protein phosphatase, Mg2+/Mn2+ dependent, 1D	-0.06
**HSAE010471**	WDYHV1	WDYHV motif containing 1	0.17
**HSAE009559**	COG6	component of oligomeric golgi complex 6	-0.11
**HSAE010643**	DCLRE1A	DNA cross-link repair 1A	-0.10
**HSAE018343**	HNRNPA3	heterogeneous nuclear ribonucleoprotein A3	-0.08
**HSAE006438**	MNS1	meiosis-specific nuclear structural 1	-0.19
**HSAE009793**	FIP1L1	FIP1 like 1 (S. cerevisiae)	-0.07
**HSAE001155**	PNISR	PNN-interacting serine/arginine-rich protein	-0.12
**HSAE000041**	STAT3	signal transducer and activator of transcription 3 (acute-phase response factor)	0.15
**HSAE004908**	DKK1	dickkopf 1 homolog (Xenopus laevis)	0.44
**HSAE030313**	Unknown	Unknown	0.20
**HSAE023444**	PNRC1	proline-rich nuclear receptor coactivator 1	-0.11
**HSAE005572**	SMAD9	SMAD family member 9	0.13
**HSAE020054**	TMEM54	transmembrane protein 54	0.14
**HSAE018904**	CDK5RAP1	CDK5 regulatory subunit associated protein 1	-0.07
**HSAE016335**	DCP2	DCP2 decapping enzyme homolog (S. cerevisiae)	-0.13
**HSAE024037**	PSMA1	proteasome (prosome, macropain) subunit, alpha type, 1	-0.08
**HSAE003487**	DLL1	delta-like 1 (Drosophila)	0.10
**HSAE004236**	AXL	AXL receptor tyrosine kinase	0.06
**HSAE024468**	TOMM5	Translocase of outer mitochondrial membrane 5 homolog (yeast)	0.06
**HSAE021697**	LRR1	leucine rich repeat protein 1	-0.14
**HSAE031910**	SEC14L1P1	SEC14-like 1 pseudogene 1	0.10
**HSAE023997**	DDX21	DEAD (Asp-Glu-Ala-Asp) box helicase 21	0.07
**HSAE001462**	EFCAB11	EF-hand calcium binding domain 11	-0.08

M, M-values were calculated as the difference between the average M-value of RIF patients and controls. Unknown, probe could not be mapped to a known gene.
